# Resilience and Related Factors: A Comparison of Fathers and Mothers of Patients With Cleft Lip and/or Palate in China

**DOI:** 10.3389/fpsyt.2021.791555

**Published:** 2022-01-13

**Authors:** Lulu Yuan, Yuqin Gao, Bochen Pan, Junyan Wang, Yanjie Wang, Caixia Gong, Weiren Wang, Xiaohan Li

**Affiliations:** ^1^Liaoning Provincial Key Laboratory of Oral Diseases, Department of Nursing, School and Hospital of Stomatology, China Medical University, Shenyang, China; ^2^School of Nursing, China Medical University, Shenyang, China; ^3^Shengjing Hospital, China Medical University, Shenyang, China; ^4^West China School/Hospital of Stomatology, Sichuan University, Chengdu, China

**Keywords:** cleft lip and/or palate, fathers, mothers, resilience, positive psychology

## Abstract

**Background:** Resilience has become a hot spot in the field of positive psychology to study life-change events. However, there were little information on resilience among the fathers and mothers of patients with cleft lip and/or palate respectively. The present study aimed to explore and compare the level and potential influential factors associated with resilience among fathers/mothers of patients with cleft lip and/or palate in China.

**Method:** A cross-sectional study was carried out between April 2019 and July 2020 among fathers/mothers of patients with cleft lip and/or palate in two cleft lip and/or palate treatment centers in China. Sixty Nine fathers and 179 mothers of patients with cleft lip and/or palate were interviewed with a questionnaire on demographic variables and the Resilience Scale-14 (RS-14), Herth Hope Index (HHI), Multidimensional Scale of Perceived Social Support (MSPSS), Revised Life Orientation Test (LOT-R), Parenting Stress Index-Short Form (PSI-SF) and Coping Health Inventory for Parents (CHIP). *T*-test/univariate one-way ANOVA, Pearson's r, hierarchical linear regression analysis were conducted to explore the influential factors of resilience.

**Results:** Fathers of patients with cleft lip and/or palate had a higher level of resilience (77.77 ± 14.18) than mothers (74.52 ± 14.33) though without significance. Resilience was positively associated with hope, perceived social support, optimism and coping and negatively correlated with parenting stress both in the fathers and the mothers. Hierarchical linear regression analysis showed that hope (β = 0.400, *P* < 0.01), coping (β = 0.281, *P* < 0.05), job status, medical payments (β = −0.240, *P* < 0.05) were found to be associated with resilience among the fathers of patients with CL/P, and all four variables in the model could explain 42.8% of the variance in resilience; Hope (β = 0.225, *P* < 0.05), perceived social support (β = 0.194, *P* < 0.05), the age of patients (β = 0.189, *P* < 0.05) were found to be associated with resilience among the mothers, and all three variables in the model could explain 27.6% of the variance in resilience.

**Conclusion:** Our study showed that, in China, fathers of patients with cleft lip and/or palate had a higher level of resilience than mothers though without significance. Hope was the only communal variable strongly associated with resilience among both the fathers and the mothers; besides, coping, job status and medical payments were found to be associated with resilience among the fathers; while perceived social support and the age of patients were found to be associated with resilience among the mothers. The results suggest that enhance hope in parents of patients with cleft lip and/or palate might greatly help improve their resilience. Besides, fathers and mothers need specific intervention to prompt their resilience.

## Introduction

Cleft lip and/or palate (CL/P) is one of the most common congenital developmental conditions in humans and the most prevalent developmental condition in the craniomaxillofacial region. Global data show that the overall incidence of cleft lip and/or palate in the world is approximately 1/700 (1). China, located in Asia, is a high-incidence area worldwide (2). China's birth surveillance data from 2000 to 2011 showed that cleft lip with or without cleft palate was the third most prevalent condition in infants in the perinatal period in China (3). Its incidence rate was approximately 1.82‰ and increased year by year (4). Cleft lip and/or palate will not only affect the patient's appearance but also adversely affect the patient's functions of pronunciation, chewing, and swallowing (5). With the development of medical treatment, the nasolabial and palate conditions of patients have greatly improved after sequential treatment. Nevertheless, there are still quite a few patients who cannot work and live like unaffected people. One important reason is that the psychological problems of the patients do not always return to normal with the improvement of shape and function (6).

The psychological problems of patients with cleft lip and/or palate are not inherent; they are the result of long-term growth and development. Ecological systems theory states that people are not independent individuals and cannot exist alone without the environment (7). Individual development should come from the interaction between the individual and the environment. The influence of the environment on individual behavior and psychological development cannot ignore. At the same time, ecological systems theory emphasizes that the growth and development of children and adolescents are affected by environmental factors. For children, especially those with chronic diseases, the most important environment comprises parents and family. Previous studies have shown that the normal development of psychological and social behavior among patients with CL/P depends mostly on the psychological state of their parents (8–10). However, parents of patients with CL/P are under more pressure, including “strike syndrome” (11–13), feeding difficulties (14, 15), difficulties seeking medical treatment (16), a lack of disease information (17), a fear of treatment failure (17), heavy financial burden of treatment (18), growth and academic problems of patients (19–22), marriage and family relationships (23, 24), stigma (25, 26), social isolation (27) and decreased quality of life (28, 29). What's more, so far, the majority of related studies are on mothers, only few on fathers. Mothers and fathers experience parenthood differently. Therefore, the psychological state of the fathers and mothers of patients with CL/P, especially the positive psychological resources, requires our attention and exploration.

Resilience is a very important concept in psychology and has been a research subject for many decades. Studies have shown that resilience is positively related to the level of mental health and negatively related to distress (30, 31). Resilience has also been found to be critical to quality of life (32). Surprisingly, however, little information about resilience in fathers and/or mothers of patients with CL/P is currently available. Considering such immense pressure on fathers and/or mothers of patients with CL/P and the importance of their positive mental resources, resilience should be approached from different perspectives, including psychological methods. Therefore, we aim to fill this knowledge gap with the current study. Furthermore, studies focusing on the relationship between resilience and other psychological factors in caregivers have shown that, in addition to demographic and clinical characteristics, hope (33, 34), social support (35, 36), optimism (37, 38), stress (39, 40) and coping (41) were all associated with resilience, but their relationship in fathers and/or mothers of patients with CL/P is unknown. The hypothesis proposes that resilience is positively associated with hope, social support, optimism and coping, and negatively associated with stress among fathers and/or mothers of patients with CL/P. Accordingly, we will test this hypothesis in the current study. We hope that the findings of our study and, in particular, the identification of influential factors of resilience may have potential value and help shed new light on the healthy development and functional balance of patients with CL/P, their parents, and their families.

## Methods

### Study Settings

This cross-sectional study was conducted in two cleft lip and/or palate treatment centers in China. Both are provincial regional treatment centers affiliated with medical universities. The first is in northeastern China, and the second is in southwestern China. Data were collected between April 2019 and July 2020. The current research was approved by the Ethical Committee of China Medical University (NO. 2018-27).

### Subjects

Respondent inclusion criteria were as follows: (1) Fathers/Mothers of patients with CL/P under 18 years old; (2) agreement to participate in the survey; (3) the capability to understand and complete the questionnaire. The exclusion criteria were as follows: (1) the respondents had other care tasks; (2) the patients had other comorbid serious diseases. The study size was arrived at by using the following formula: $n=\frac{Z_{\alpha }^{2}\sigma^{2}}{\delta^{2}}$ The parameters were α = 0.05, Zα = 1.96, σ = 15.39(arrived via the pre-test), and δ = 2; therefore, *n* = 1.96^2*^15.39^2^/2^2^ = 227.47. Considering that there were invalid questionnaires, the sample size was increased by 10~20%, and the final sample size was 251~273.

### Data Collection

The whole process of the study was anonymous and voluntary for the parents. The investigators consisted of four nurses who were trained uniformly by the researcher. At the time of the patient's visit, informed consent was given to their parents. After agreeing to participate, one of the parents filled out a paper version of the questionnaire in a separate, undisturbed space in the hospital, so that the parents would not influence each other in completing the questionnaires. The investigators were responsible for providing explanations for questionnaire items without any inducement. Another trained investigator conducted quality control on the spot and then collected the questionnaires. Epidata software (version 3.1) was used for data entry and double-checking.

### Tools

Demographic and clinical characteristics were collected via a general questionnaire. Demographic characteristics consisted of the ages of patients and fathers/mothers, the sex of patients, whether the patient was an only child, developmental status of patients, education level of fathers/mothers, job status of fathers/mothers, religious belief of fathers/mothers, monthly income, residence area, family structure, and medical payments. Clinical variables included patient's type of oral cleft and family history (Whether there is a cleft lip or palate in the family).

#### Measurement of Resilience

Respondents' resilience was measured with the Resilience Scale-14 (RS-14) (42). The RS-14 includes 14 items, and each item is rated on a 7-point scale, with a total score ranging from 14 to 98. The Chinese version of the RS-14 has been used in previous studies, and the reliability and validity were confirmed (30). Cronbach's alpha coefficient for the total scale of resilience was 0.901 in the present study.

#### Measurement of Hope

Hope was assessed by the Herth Hope Index (HHI) (43), which contains 3 subscales: temporality and future, positive readiness and expectancy, and interconnectedness. The HHI consists of 12 items, and each item is scored on a 4-point scale. The total HHI score ranges from 12 to 48, and a higher total score reflects a higher level of hope. The Chinese version of the HHI has been found to have good reliability and validity (44). In the current study, Cronbach's α was 0.854.

#### Measurement of Social Support

The level of perceived social support was assessed by the Chinese version of the Multidimensional Scale of Perceived Social Support (MSPSS) (45), which measures perceived support from three social relationships: fam-ily, friends and significant others (such as relatives and colleagues). The MSPSS includes 12 items rated on a 7-point scale. The total score ranges from 12 to 84, with a higher score indicating higher social support. The scale had good reliability and validity among various Chinese populations (46, 47). In this study, Cronbach's α of the MSPSS was 0.928.

#### Measurement of Optimism

Optimism was assessed by the 10-item Revised Life Orientation Test (LOT-R), which was designed by Scheier et al. (48). The LOT-R consists of ten items using a 5-point rating system, three of which are for optimism, three of which are for pessimism, and the other four items serve as fillers. A higher score indicates a higher level of optimism. The LOT-R has shown good reliability and validity among various Chinese populations (47). Cronbach's α was 0.600 in the current research.

#### Measurement of Parenting Stress

Parenting stress was assessed by the Parenting Stress Index-Short Form (PSI-SF) (49), which contains 3 subscales: parent distress, parent-child dysfunctional interaction, and difficult child. The PSI-SF consists of 36 items, and each item is scored using a 5-point scale, with a total score ranging from 36 to 180. Higher scores indicate a higher level of parenting stress. The Chinese version has demonstrated good reliability and validity (50). Cronbach's α was 0.940 in this study.

#### Measurement of Coping

The Coping Health Inventory for Parents (CHIP) was developed by Mc Cubbin (51) to measure what coping strategies respondents used to maintain normal family life when there was a child with chronic disease in the family. The CHIP is a 45-item scale rated on a 5-point scale, with a total score ranging from 45 to 225. Higher scores indicate a higher level of coping. The scale has been widely used among the Chinese population (52). Cronbach's α was 0.940 in the current study.

### Statistical Analyses

Statistical Package for Social Sciences (SPSS 22.0 for Windows) was used to conduct data analyses. Significance for all statistical tests was set at 0.05 (2-tailed). The normality and homogeneity of variances were first tested for each continuous variable. Independent sample *T*-tests and one-way ANOVA were used to describe distributions of resilience in categorical demographic and clinical variables among fathers and mothers. Pearson's r test was conducted to test the correlations among hope, perceived social support, optimism, parenting stress, coping and resilience. Hierarchical linear regression analyses were conducted to test the study hypotheses. The variables with *P* < 0.2 in one-way ANOVA/*t*-test were entered into step 1 of the hierarchical regression analysis as control variables in order to not overfit the regression models (53). Then, the independent variables (hope, perceived social support, optimism, parenting stress, and the coping of the parents) were also entered into step 2 of the hierarchical regression. Variables were entered in the regression analysis at *P* < 0.05 and removed from the model at *P* > 0.10. Multicollinearity diagnostic tests were carried out by the Tolerance and variance inflation factor (VIF). Data provided in the regression models included the standardization regression coefficient (β), R^2^, adjusted R^2^ (Adj. R^2^), R^2^-change and F value.

## Results

### Descriptive Statistics

In the current study, 262 questionnaires were distributed. Among them, seven participants refused to attend the survey, seven questionnaires were considered invalid. The number of valid questionnaires was 248, yielding an effective response rate of 94.66%.

Among the 248 participants, 69 (27.8%) were fathers, and 179 (72.2%) were mothers. The mean age of the patients was 7.70 years (SD = 5.76, range 0.08–18). In terms of the clinical variables, among all the patients, 53 (21.4%) had cleft lip, 77 (31%) had cleft palate and 118 (47.6%) had both cleft lip with cleft palate. Only 13 (5.2%) patients had a family history of cleft lip and/or palate. The demographic and clinical characteristics and the level of resilience among fathers/mothers of patients with CL/P were described in Table 1.

**Table 1:** Demographic and clinical characteristics and the level of resilience among fathers and mothers of patients with CLP (*n* = 248).

**Variables**	**Fathers (*****N*** **=** **69)**				**Mothers (*****N*** **=** **179)**			
	***N* (%)**	**Mean (SD)**	**T/F**	***P*-value**	***N* (%)**	**Mean (SD)**	**T/F**	***P*-value**
**Sex of patients**			0.220	0.827			0.732	0.465
Male	42 (60.9)	78.07 (12.88)			94 (52.5)	75.27 (13.02)		
Female	27 (39.1)	77.30 (16.26)			85 (47.5)	73.69 (15.70)		
**Is the patient an only child?**			0.905	0.369			−0.582	0.561
Yes	23 (33.3)	79.96 (10.85)			73 (40.8)	73.77 (15.27)		
No	46 (66.7)	76.67 (15.58)			106 (59.2)	75.04 (13.71)		
**Development condition of patients**			0.596	0.554			0.160	0.852
Normal	56 (81.2)	78.14 (14.14)			146 (81.6)	74.77 (14.33)		
Slower	10 (14.5)	74.00 (14.41)			27 (15.1)	73.07 (15.25)		
Faster	3 (4.3)	83.33 (16.80)			6 (3.3)	74.83 (11.62)		
**Age of fathers/mothers**			0.450	0.640			0.547	0.580
≤ 25	1 (1.4)	73.00			12 (6.7)	76.08 (13.81)		
26–45	59 (85.5)	77.24 (14.44)			143 (79.9)	73.97 (14.400		
>46	9 (13.1)	81.78 (13.23)			24 (13.4)	77.04 (14.47)		
**Education of fathers/mothers**			0.494	0.612			3.010	0.052
Middle school or lower	32 (46.4)	76.06 (12.46)			98 (54.7)	73.07 (15.31)		
High school or secondary school	17 (24.6)	78.29 (12.58)			39 (21.8)	73.10 (12.51)		
College or university or above	20 (29.0)	80.05 (17.96)			42 (23.5)	79.21 (12.76)		
**Job status**			3.031	0.036			0.441	0.724
Regular employee	35 (50.7)	82.26 (9.68)			73 (40.8)	75.92 (14.06)		
Temporary employee	21 (30.4)	71.57 (18.50)			41 (23.0)	73.51 (14.84)		
Resigned for the child	5 (7.2)	72.00 (13.00)			14 (7.8)	72.21 (15.70)		
Unemployed	8 (11.6)	78.00 (13.57)			51 (28.5)	73.96 (14.18)		
**Religious belief**			0.498	0.620			−1.074	0.284
No	63 (91.3)	78.03 (14.48)			157 (87.7)	74.09 (14.59)		
Yes	6 (8.7)	75.00 (11.28)			22 (12.3)	77.59 (12.23)		
**Income (RMB, yuan)**			2.705	0.053			0.539	0.656
<3,000	26 (37.7)	72.58 (17.21)			69 (38.5)	73.81 (14.93)		
3,000–5,000	26 (37.7)	78.46 (12.46)			75 (42.0)	75.99 (13.75)		
5,000–10,000	13 (18.8)	84.54 (8.05)			28 (15.6)	73.25 (11.41)		
>10,000	4 (5.8)	85.00 (5.72)			7 (3.9)	70.86 (24.38)		
**Residence**			1.687	0.096			0.996	0.321
Urban	26 (37.7)	81.42 (17.17)			68 (38.0)	75.88 (14.30)		
Rural	43 (62.3)	75.56 (11.70)			111 (62.0)	73.68 (14.35)		
**Family structure**			0.337	0.798			0.563	0.640
Single-parent family	5 (7.3)	80.80 (13.29)			9 (5.0)	71.78 (18.69)		
Two-parent family	26 (37.7)	77.85 (12.48)			86 (48.0)	74.03 (14.90)		
Extended family	37 (53.6)	77.65 (15.70)			80 (44.7)	75.66 (13.53)		
Step-family	1 (1.4)	65.00			4 (2.3)	68.25 (5.56)		
**Medical payments**			2.146	0.035			−1.840	0.068
Public welfare program	27 (39.1)	82.22 (10.38)			82 (45.8)	72.39 (15.82)		
Others	42 (60.9)	74.90 (15.61)			97 (54.2)	76.32 (12.76)		
**Family history**			0.567	0.572			0.222	0.825
Yes	3 (3.3)	82.33 (18.50)			10 (5.6)	75.50 (16.11)		
No	66 (95.7)	77.56 (14.10)			169 (94.4)	74.46 (14.27)		
**Type of oral cleft**			1.662	0.198			0.241	0.786
Cleft lip	11 (15.9)	76.55 (11.40)			42 (23.5)	75.86 (14.40)		
Cleft palate	28 (40.6)	74.57 (17.84)			49 (27.4)	74.27 (16.71)		
Cleft lip and palate	30 (43.5)	81.20 (10.39)			88 (49.1)	74.02 (12.95)		

*Red represents the P-value of the variable < 0.05 and the blue P < 0.2. The colors can be removed if necessary*.

*N, number*.

### Resilience Level

Although there was no significant difference in the level of resilience between fathers and mothers of patients with CL/P, the resilience level of fathers was higher than that of mothers, which was were described in Table 2.

**Table 2:** Level and comparison of resilience between fathers and mothers.

	***N***	**Mean ± SD**	**T**	***P-*value**
Fathers	69	77.77 ± 14.18	1.604	0.11
Mothers	179	74.52 ± 14.33		

### Correlation Among Continuous Variables

The results of the correlation analysis of hope, perceived social support, optimism, parenting stress, and coping with resilience among **fathers** of patients with CL/P are presented in Table 3. Resilience was positively associated with hope (r = 0.533, *p* < 0.001), perceived social support (r = 0.325, *p* < 0.01), optimism (r = 0.261, *p* < 0.01) and coping (r = 0.462, *p* < 0.001) and negatively correlated with parenting stress (r = −0.312, *p* < 0.01).

**Table 3:** Descriptive statistics and correlations in continuous variables among fathers (*n* = 69).

	**Means**	**SD**	**Resilience**	**Hope**	**Social support**	**Optimism**	**Parenting stress**
Resilience	77.77	14.18	1				
Hope	40.16	4.84	0.533***	1			
Perceived social support	65.26	12.65	0.325**	0.447***	1		
Optimism	16.65	2.97	0.261**	0.413*	0.292**	1	
Parenting stress	84.91	20.60	−0.312**	−0.496***	−0.433***	−0.340**	1
Coping	170.12	25.34	0.462***	0.443***	0.505***	0.108	−0.422***

*P < 0.05; **P < 0.01; ***P < 0.001 (two-tailed).

The results of the correlation analysis of hope, perceived social support, optimism, parenting stress, and coping with resilience among **mothers** of patients with CL/P are presented in Table 4. Resilience was positively associated with hope (r = 0.448, *p* < 0.001), perceived social support (r = 0.420, *p* < 0.01), optimism (r = 0.226, *p* < 0.01) and coping (r = 0.357, *p* < 0.001) and negatively correlated with parenting stress (r = −0.382, *p* < 0.001).

**Table 4:** Descriptive statistics and correlations in continuous variables among mothers (*n* = 179).

	**Means**	**SD**	**Resilience**	**Hope**	**Social support**	**Optimism**	**Parenting stress**
Resilience	74.52	14.33	1				
Hope	38.61	4.56	0.448***	1			
Perceived social support	63.58	12.43	0.420***	0.504***	1		
Optimism	16.58	2.62	0.226**	0.363***	0.330***	1	
Parenting stress	84.16	22.11	−0.382***	−0.573***	−0.466***	−0.388***	1
Coping	167.93	25.06	0.357***	0.491***	0.343***	0.219**	−0.395***

**P < 0.01; ***P < 0.001 (two-tailed).

### Hierarchical Linear Regression Analysis

Hierarchical linear regression analysis was conducted to identify the influential factors of resilience among the fathers/mothers of patients with CL/P. Variables that were significantly associated with resilience in the univariate analyses, and variables related to the psychology of parents were included in the multiple regression analysis.

Among **fathers**, demographic variables (the age of patients, job status, income, residence, medical payments, and type of oral cleft), hope, perceived social support, optimism, parenting stress, and coping were included in the regression analysis. The results of the analysis are shown in Table 5. Hope (β = 0.400, *P* < 0.01), coping (β = 0.281, *P* < 0.05), job status, medical payments (β = −0.240, *P* < 0.05) were found to be associated with resilience among the fathers of patients with CL/P, and all four variables in the model could explain 42.8% of the variance in resilience.

**Table 5:** Hierarchical linear regression analysis on results of resilience among fathers of patients with CL/P (*n* = 69).

**Variables**	**Resilience**					
	**β**	***P*-value**	**β**	***P*-value**	**Tolerance**	**VIF**
**Step 1**						
Age of patients	0.031	0.819	0.047	0.686	0.616	1.623
Job status of fathers						
Dummy_1	−0.066	0.752	−0.068	0.707	0.259	3.865
Dummy_2	−0.299	0.099	−0.317	0.043*	0.359	2.785
Dummy_3	−0.271	0.065	−0.220	0.079	0.558	1.791
Residence	0.026	0.853	0.093	0.457	0.543	1.840
Income	0.308	0.038*	0.214	0.102	0.511	1.956
Medical payments	−0.318	0.013*	−0.240	0.029*	0.739	1.353
Type of oral cleft						
Dummy_1	−0.006	0.969	−0.090	0.542	0.391	2.555
Dummy_2	0.102	0.568	0.073	0.637	0.352	2.842
**Step 2**						
Hope			0.400	0.002**	0.548	1.823
Optimism			0.047	0.681	0.644	1.553
Social support			−0.006	0.961	0.601	1.665
Parenting stress			0.154	0.249	0.484	2.068
Coping			0.281	0.021*	0.603	1.657
F	2.749**		4.629***			
R^2^	0.295		0.545			
adjR^2^	0.188		0.428			
R^2^-change	-		0.250			

***P < 0.001 (two-tailed); **P < 0.01 (two-tailed); *P < 0.05 (two-tailed).

Among **mothers**, demographic variables (the age of patients, education, and medical payments), hope, perceived social support, optimism, parenting stress, and coping were included in the regression analysis. The results of the analysis are shown in Table 6. Hope (β = 0.225, *P* < 0.05), perceived social support (β = 0.194, *P* < 0.05), the age of patients (β = 0.189, *P* < 0.05) were found to be associated with resilience among the mothers of patients with CL/P, and all four variables in the model could explain 27.6% of the variance in resilience.

**Table 6:** Hierarchical linear regression analysis on results of resilience among mothers of patients with CL/P (*n* = 179).

**Variables**	**Resilience**					
	**β**	***P*-value**	**β**	***P*-value**	**Tolerance**	**VIF**
**Step 1**						
Age of patients	0.150	0.086	0.189	0.014*	0.702	1.424
Education of mothers						
Dummy_1	0.067	0.424	0.039	0.594	0.748	1.336
Dummy_2	0.243	0.007**	0.108	0.177	0.635	1.575
Medical payments	0.102	0.173	0.084	0.203	0.941	1.063
**Step 2**						
Hope			0.225	0.012*	0.520	1.922
Optimism			−0.012	0.865	0.789	1.267
Social support			0.194	0.014*	0.663	1.509
Parenting stress			−0.124	0.140	0.579	1.727
Coping			0.132	0.083	0.706	1.416
F	2.874**		8.530***			
R2	0.062		0.312			
adjR2	0.040		0.276			
R2-change	-		0.250			

***P < 0.001 (two-tailed); **P < 0.01 (two-tailed); *P < 0.05 (two-tailed).

## Discussion

The current study explored the level and influential factors of resilience among fathers and mothers of patients with CL/P.

### Resilience Levels Among Fathers and Mothers of Patients With CL/P

The level of resilience in the study was similar to (54) or higher (55) than previous studies of caregivers of children with chronic diseases. Besides, the resilience level of fathers was higher than that of mothers, though there was no significant difference. The finding was significant due to the lack of information available in the literature about resilience in parents of patients with CL/P. This result is explainable. On one hand, resilience emphasizes that individuals can actively respond and adapt well in the face of negative events such as threats, adversity, or pressure (56–58), in which fathers have certain advantage over mothers. As parents accept and work hard on the disease gradually, their resilience level will become higher. On the other hand, CL/P is not a life-threatening disease but a disease with a promising cure. The patient's condition will improve as the treatment progresses. Therefore, in our clinical work, we should pay more special attention to the psychological status of parents of young patients.

At the same time, we noticed that more mothers (179, 72.2%) were recruited in the study than fathers (69, 27.8%). The result came as no surprise to us, because quite many studies have shown the similarly information, both in parents of patients with CL/P (24, 59) and families having a child with chronic conditions (60). This phenomenon resulted from the fact that mothers usually took more responsibilities on child caring in families in China, in comparison with fathers. What's more, more fathers were employed than mothers in this study, similar to other research (12, 61). Besides, mothers had higher levels of parental involvement behaviors than fathers (62).

### Factors Associated With Resilience Among Fathers and Mothers of Patients With CL/P

In the current study, hope, coping, job status and medical payments were found to be associated with resilience among the **fathers** of patients with CL/P. Hope, perceived social support and the age of patients were found to be associated with resilience among the **mothers** of patients with CL/P.

According to the results of logistic regression analysis, hope was the **only** communal variable strongly associated with resilience among **both** fathers and mothers of patients with CL/P, which was similar to the results in previous studies on the resilience of caregivers of children with chronic diseases (63, 64). Hope has been confirmed to be related to almost all health outcomes (65). It is part of the psychological capitals of caregivers. Hope is an important resilience factor when caregivers cope with the care pressure of patients, which can help caregivers alleviate negative psychological distress. Lloyd's research found that as an influential factor of mental resilience, a low level of hope could predict the anxiety and depression level of parents of children with intellectual disabilities (34). In addition, hope is an important influencing factor in the development of caregivers' resilience. In a longitudinal study, hopefulness was found to be capable of predicting resilience among hereditary colorectal cancer patients (66). Therefore, enhancing the level of hope may become an important strategy to increase the level of resilience among both fathers and mothers of patients with CL/P in China.

In addition to hope, we found that coping was another positive psychological resource for the resilience among **fathers** of patients with CL/P. There were rare studies on resilience of fathers. Fortunately, this result was consistent with previous findings on the psychological conditions among caregivers and mothers of patients with CL/P (67, 68). Active coping strategies are the behaviors taken by caregivers under the condition of good psychological adjustment, and the implementation of active coping strategies, in turn, will help to improve the psychological condition of themselves. Then, a virtuous circle forms, and the growth of parents' psychological capital is prompted. Nadia Hasanzadeh's study on mothers of patients with CL/P confirmed this and further pointed out that positive coping strategies not only help improve the mother's psychological status but also benefit the mother's parent-child behavior (68). Therefore, active coping strategies should be valued, and we suggest that in clinical care, clinicians and nurses should help fathers of patients with CL/P develop active coping plans to adjust their psychological pressure and relieve negative emotions.

In this study, we also found that perceived social support made a positive contribution to the resilience of **mothers** of patients with CL/P. The positive effect of social support has been confirmed in many studies, both studies on the resilience of caregivers with chronic diseases (35, 69) and studies on psychological conditions of caregivers of patients with CL/P (12, 67, 70). Perceived social support is a part of the positive psychological capital of almost all patients and caregivers. People with higher levels of perceived social support have more resources they can use to care for the patients, which lightens the burden of care and psychological burden. The level of resilience and psychological status are naturally improved. However, it is worth noting the results of two qualitative studies (35, 36). Their findings showed that the support of family and friends was not always sufficient to promote the psychological resilience of caregivers, and the support function helped to enhance resilience only when it was considered to match the needs. Therefore, the specific role of perceived social support needs further study and discussion.

On the other hand, it is interesting to note that some of the demographic variables have been demonstrated specifically associated with the resilience of parents of patients with CL/P.

First, we found that job status and medical payments were of great significance for the resilience of the fathers. In the study, fathers with regular employee had the highest level of resilience than other status. Regular employee meant a stable income. Besides, fathers of patients with public welfare program had higher level of resilience than others. Having public welfare program could be interpreted as less financial pressure. In other words, these two variables related to the fathers' resilience suggests that the fathers bear more economic pressure in the treatment of children. This is in line with the role of fathers in most Chinese families.

Finally, it was not surprising to find that the age of patients was an important factor for the resilience of the mothers. This has been confirmed not only in the study of the resilience of caregivers of children with chronic diseases (71) but also in the study of the psychological conditions of caregivers of patients with CL/P (28). In the study, the age of patients was associated with resilience of the mothers, not the fathers, possibly for the primary caregivers for CL/P was often the mothers. The resilience of mothers increases with the age of patients. It is a good phenomenon, mainly because as the patient grows up and the treatment progresses, the mothers can take care of their children more easily. However, one problem that we cannot ignore is the patient's psychological and developmental problems. The improvement of clinical symptoms does not mean the complete end of the child's treatment. In clinical practice, we have also found that many parents of patients with CL/P pay too much attention to the improvement of their children's appearance and function while ignoring their children's performance in interpersonal communication, personality development and school achievement and attribute the patients' inadaptability in these aspects only to different personalities and characteristics. Previous studies have also found similar problems (72–74). In summary, although the burden of life care and medical care for parents is reduced as patients with CL/P grow up, we should still remind parents and provide parents with relevant professional services to promote the healthy growth of patients' psychology and society.

However, the current study results were partially inconsistent with our hypothesis in that optimism and parenting stress showed no significant relations neither with fathers nor mothers. Therefore, further research is still needed to explore the exact mechanism of the two variables.

## Strength and Limitations

The current study aimed to identify the potential influencing factors associated with resilience in fathers and mothers of patients with CL/P. In this respect, our study has added some new information. The results provided evidence that hope was the only communal variable strongly associated with resilience among both the fathers and the mothers; coping, job status and medical payments were found to be associated with resilience among the fathers; while perceived social support and the age of patients were found to be associated with resilience among the mothers. In addition, our study provided clinical information for the definition of resilience. The results of our study showed that the age of patients was a vital factor for the resilience of the mothers. This result indicated that resilience may be a developable quality for dealing with adversity.

Due to the cross-sectional design, the causal relationship could not be confirmed. Future studies should be carried out to assess whether interventions could improve the level of resilience among fathers and mothers of patients with CL/P. In addition, we focused only on the associations of resilience with hope, optimism, parenting stress, coping and perceived social support; other factors that may be important to consider for resilience were not included. Besides, the long number of questions may represent a limitation for the quality of response. Moreover, a larger sample is needed to improve the representativeness. Despite some limitations, our study provided important new information on resilience in parents of patients with CL/P, and it had useful theoretical and clinical implications.

## Conclusions

Our study showed that, in China, fathers of patients with CL/P had a higher level of resilience than mothers though without significance. Hope was the only **communal** variable strongly associated with resilience among both fathers and mothers of patients with CL/P; besides, coping, job status and medical payments were found to be associated with resilience among the **fathers**; while perceived social support and the age of patients were found to be associated with resilience among the **mothers** of patients with CL/P.

## Data Availability Statement

The raw data supporting the conclusions of this article will be made available by the authors, without undue reservation.

## Ethics Statement

The studies involving human participants were reviewed and approved by Committee on Human Experimentation of China Medical University (2018-27). The patients/participants provided their written informed consent to participate in this study.

## Author Contributions

LY and XL were responsible for conception and design of the study. YG gave directions to the study. LY, JW, YW, and CG performed data extraction. LY did the data analysis and wrote the manuscript. BP and WW contributed to the revision of the manuscript. All authors have reviewed the manuscript and given final approval of the version to be published.

## Funding

This study was funded by the project of Education Department of Liaoning Province (LJKR0281) and the project of College of Nursing, China Medical University (2017HL-17). The funding bodes played no role in the design of study, collection, analysis and interpretation of data, or in writing the manuscript.

## Conflict of Interest

The authors declare that the research was conducted in the absence of any commercial or financial relationships that could be construed as a potential conflict of interest.

## Publisher's Note

All claims expressed in this article are solely those of the authors and do not necessarily represent those of their affiliated organizations, or those of the publisher, the editors and the reviewers. Any product that may be evaluated in this article, or claim that may be made by its manufacturer, is not guaranteed or endorsed by the publisher.
